# Determinants of life expectancy at birth: a longitudinal study on OECD countries

**DOI:** 10.1007/s10754-022-09338-5

**Published:** 2022-11-11

**Authors:** Paolo Roffia, Alessandro Bucciol, Sara Hashlamoun

**Affiliations:** 1 Department of Business Administration, University of Verona, Polo S. Marta, Via Cantarane 24, 37129 Verona, Italy; 2 Department of Economics, University of Verona, Polo S. Marta, Via Cantarane 24, 37129 Verona, Italy

**Keywords:** Life expectancy, Health-care system, Health expenditure, OECD countries, COVID-19, I12, I18, C23

## Abstract

This paper analyses the influence of several determinants on life expectancy at birth in 36 OECD countries over the 1999–2018 period. We utilized a cross-country fixed-effects multiple regression analysis with year and country dummies and used dynamic models, backward stepwise selection, and Arellano–Bond estimators to treat potential endogeneity issues. The results show the influence of per capita health-care expenditure, incidence of out-of-pocket expenditure, physician density, hospital bed density, social spending, GDP level, participation ratio to labour, prevalence of chronic respiratory diseases, temperature, and total size of the population on life expectancy at birth. In line with previous studies, this analysis confirms the relevance of both health care expenditure and health care system (physicians and hospital beds in our analysis) in influencing a country’s population life expectancy. It also outlines the importance of other factors related to population behaviour and social spending jointly considered on this outcome. Policy makers should carefully consider these mutual influences when allocating public funds, particularly after the COVID-19 pandemic period.

## Introduction

Over the past two centuries, one of the extraordinary achievements in developed countries has been the remarkable increase in life expectancy. Life expectancy at birth is the average number of years that a person can be expected to live from birth, supposing constant age–specific mortality levels (Laranjeira & Szrek, 2016). It is probably one of the most important indicators of a country’s well-being (Ho & Hendi, 2018), a proxy for health that the World Health Organization (WHO, 2006) defines as “a state of complete physical, mental and social well-being and not merely the absence of disease or infirmity”. Life expectancy is something more than just a figure; indeed, it is the way to understand and appraise the effects of government policies, human behaviour, and cultural patterns in a given context or country. Life expectancy could influence many other social and economic aspects such as fertility rate, consumer propensity to spend, human capital investment, pension expenditure, public finance and economic growth (Shaw et al., 2005). Indeed, health improvements produce economic growth (Bloom & Canning, 2000; Schultz, 2002) as a higher life expectancy expands investments in many fields like innovation and production (Cervellati & Sunde, 2013; Prettner, 2013). Nearly all studies investigating economic growth found a positive correlation between population health measured by life expectancy and the economy (Bhargava et al., 2001; Sunde & Vischer, 2015).

The new generation of babies born since 2000 is predicted to celebrate their 100th birthday and their children may even live longer (Christensen et al., 2009). Although at the end of World War II the main reason for an improvement in longevity was related to lower infant mortality, in recent years a higher life expectancy has been achieved through better living conditions that improved the survival prospects of older adults, especially those over 65 (Aisa et al., 2014). This trend has been proven in all OECD countries albeit often in a non-linear trend and with country-specific situations. For example, in the Netherlands, after 20 years of a slow increase between the 1980s and 1990s, life expectancy experienced a sudden boost in 2002 and increased by almost 2 years, reaching 82 years for women and 78 for men (Mackenbach et al., 2011), thanks to an increase in health care for the elderly and a relaxation in budgetary constraints. Conversely, between 2014 and 2015 another eighteen high-income countries^1^ registered an average decline of 0.21 years for women and 0.18 years for men (mostly referred to adults over 65 years). This phenomenon was probably due to the high incidence of cardiovascular and/or respiratory diseases and a more severe influenza season (Ho & Hendi, 2018). During the same period, the United States experienced a similar decline that involved younger adults, which some scholars attributed to external causes like drug abuse (Ho & Hendi, 2018). Although episodes of yearly decreases and sudden accelerations have occurred in almost all the countries, life expectancy seems to have a long-term positive trend. In the new millennium, excluding the 2020–2021 effects of the COVID-19 pandemic crisis, global longevity has increased by 5.5 years, the fastest increase since the 1960s. Regarding countries belonging to the OECD, life expectancy at birth increased from 72 years in 1950 to over 75 years in 2015 (Christensen et al., 2009), with peaks over 80 years in 2018 for most European countries.

In light of the above reported considerations, is it still possible to further enhance life expectancy at birth? Is so, how could governments or groups of individuals intervene to improve life expectancy? In other words: Is there a way for public spending to achieve that targeted improvement? Quite simply, life expectancy at birth depends on two main determinants: the chance of surviving the early years and, mainly, the type of behaviour engaged in and the external support system that people have access to Adler and Newman 2002); Braveman & Gottlieb, 2014). Health care expenditure and a country’s health care system are powerful factors that influence both determinants (Nixon & Ulmann, 2006). In all developed countries, due to constitutional rights related to safeguarding the population, the health care system is a typical public issue associated with public spending (Heuvel & Olaroiu, 2017; Papanicolas et al., 2019). In recent years, in many Western countries, before the COVID-19 pandemic period, public health care expenditure suffered budget constraints in a wider revision plan of the “welfare state” (Aisa et al., 2014; Okunade & Suraratdecha, 2000). According to economic principles, public spending should be oriented towards maximum efficiency, given a certain level of effectiveness that we expect from health care system outcomes (surgery, specialised medicine, preventive medicine). Health care outcomes in association with behavioural, social and other factors could influence life expectancy (Braveman & Gottlieb, 2014; Dahlgren & Whitehead, 1991). Given the interrelationships and their mutual interdependence, we believe that a “holistic” approach which considers these variables together in one single model and analyses their influence in a large sample of countries over a long period best suits this cognitive purpose (Shaw et al., 2005).

In our study, we aimed to address this supposed relationship by adopting a wide perspective. We considered 36 out of the 38 OECD countries (unfortunately social spending data are missing for Colombia and Costa Rica) over the last twenty years before the COVID-19 pandemic (1999–2018) and created a panel dataset for our analysis by collecting data from several sources. We contribute to the existing literature in three ways. First, using an econometric model, we identified a wide set of variables related to health care, social and behavioural issues, which explain most of the life expectancy trend in recent years. Second, we extended the analysis to a wide set of variables and posited the importance of both health care system outputs and other social, behavioural or structural variables in an integrated view. Third, we contributed by supporting the strengthening of both health care systems (physicians and hospital beds) and social care systems as the two relevant drivers associated with life expectancy at birth and, therefore, population health in general.

The paper is structured as follows: Section “Literature review and research hypotheses” provides a literature review and reports our research hypotheses. Section “Data and model specification” describes the data used and the models involved. Section Results shows and comments on the results. Lastly, Sect. “Conclusion, limitations and future research” contains conclusions, limitations and outlines some implications of the present study.

## Literature review and research hypotheses

Focusing on the health status of a given population, scholars proposed input–output models where variables such as life expectancy at birth, life expectancy at 65 years, healthy life expectancy for the total population and by gender, or mortality indicators (mortality rate, infant mortality, potential years of life lost) were identified as good proxies for “health” and acted as dependent variables (Cochrane et al., 1978; Jaba et al., 2014). In these studies, some models assigned to the health care system (HCS) an active role of transforming certain inputs (such as medical materials, labour, machinery) to outputs such as visits, surgery, therapies, etc. (Cochrane et al., 1978; Papanicolas et al., 2019). Regressors were mainly identified in variables related to the health care system and its inputs or to lifestyle and global health of the population. The most widely used dependent variable was health expectancy at birth (Ho & Hendi, 2018; Nolte et al., 2002), which was the result of HCS effectiveness plus the influence of other factors (Park & Nam, 2018; Ranabhat et al., 2018) such as metabolic disorders, cardiovascular diseases, cancer propensity, respiratory diseases, life style behaviours (diet, or physical activity), working conditions, environmental factors (pollution, CO_2_ emissions, water purity), and social support (pension funds or other social spending) (Braveman & Gottlieb, 2014; Dahlgren & Whitehead, 1991; Lobb, 2009; Nixon & Ulmann, 2006).

From the literature we identified seven categories as potential determinants of life expectancy: (1) health care expenditures, (2) health financing policies, (3) elements of medical care, (4) health habits and population health, (5) social determinants, (6) social spending, and (7) other external factors. Each category is discussed below.

### Health care expenditures

Over the last decades, scholars have pointed out a positive influence of resources employed in a health care system and the longevity of the population (Jaba et al., 2014; Nixon & Ulmann, 2006). In these studies, health care expenditure was measured in both absolute terms (per capita expenditure) and relative terms (e.g., share of GDP spent on health care). Increases in health care spending with an expansion of health care services, especially for the elderly, have been found to be associated with a rise in longevity (Mackenbach et al., 2011). Although some scholars argued that health outcomes were not directly impacted by health care expenditures (Barlow & Vissandjée, 1999; Blázquez-Fernández et al., 2018; Rhee, 2012), most of the studies in the 1970s, 1990s and 2000s showed a positive relationship between the two terms and included some forms of health expenditure as inputs in their models (Berger & Messer, 2002; Cochrane et al., 1978; Crémieux et al., 1999, 2005).

Focusing only on public expenditure, high levels were associated with higher life expectancy (Aisa et al., 2014; Linden & Ray, 2017), whereas inequalities in this type of spending accounted for different health care system outcomes (Jaba et al., 2014). According to the OECD, health spending has been the major driver for longevity gains in recent decades, as a 10% increase in per capita health expenditure is associated with a gain of 3.5 months in life expectancy (Papanicolas et al., 2019). Cross-country comparisons have confirmed this relationship, even if there are some outlier countries such as the United States, which has high spending associated with lower life expectancy. Indeed, a health care system performs better (and therefore promotes higher life expectancy) if it generates more outputs (health outcomes) for a given level of inputs, or if it obtains the same outcomes with fewer resources (Elola et al., 1995). For the above reasons, we formulate the following research hypothesis:

#### HP1

*Total health care expenditure is positively correlated to population life expectancy.*

### Health financing policies

Health financing policies refer to resources that are allocated to cover population health needs. The aim of a HCS is to make funding available to providers and to ensure that all individuals have access to public and personal health care, thereby avoiding financial challenges. To make sure that countries achieve universal health coverage, private health expenditures should be reduced, encouraging pre-paid funds (health taxes) to support health systems.

Since 1978, the WHO has stressed the importance of having a health care system oriented towards universal health care coverage in order to improve life expectancy, wealth, economic development (Dye et al., 2013) and greater economic growth (Ranabhat et al., 2018).

Politicians and policy analysts advocating for universal health care coverage over the past decades have facilitated the implementation of more inclusive health care systems and health policies (Lee, 2003). Indeed, in recent decades laws in OECD countries have allowed them to achieve more than 90% health coverage, which is the level currently considered “universal” for the population. The only exception (excluding Mexico and Chile, recent OECD members) is the United States where a significant proportion of the population has no health coverage yet (Moreno-Serra & Smith, 2014). Auspicated universal coverage does not mean that citizens are exonerated from paying for all their health expenses, particularly when they decide to seek extra, specialized care or when they buy over-the-counter medicine. The out-of-pocket expenditure accounts for these expenses and is usually reported as a percentage of the overall health expenditure. Ranabhat et al. (Ranabhat et al., 2018), in a study of more than 180 countries, found a negative influence of out-of-pocket expenditure on life expectancy. Rhee (Rhee, 2012) pointed out that when public and private health expenditures are jointly included, the latter appears to be less significant. By contrast, Aisa et al. (Aisa et al., 2014), regarding public health expenses in relation to life expectancy, found an inverted U-shaped curve. Accordingly, Berger and Messer (Berger & Messer, 2002) in a sample of 20 OECD countries reported that the mortality rate increased when health care expenditures are covered by public financing. In Europe, especially in countries like Italy and Spain that have two of the highest life expectancies in the world, out-of-pocket payments seem to have increased over time, underlining the importance of private initiatives in the provision of health care (Grima et al., 2018). In OECD countries, out-of-pocket payments may still be considered a burden that creates access barriers to health care (Galbraith et al., 2005). Considering the previous analysis, we posit the following research hypothesis:

#### HP2

*Individual contributions to health financing, measured by out-of-pocket payments, is associated to life expectancy.*

### Elements of medical care

Health care expenditure is the value of the resources allotted for health processes in both general and specialized medicine. Previous studies observed a positive relationship between life expectancy and an increase in the number of physicians and hospital beds (Nixon & Ulmann, 2006). Number of physicians and hospital beds are frequently used as proxies for health care processes output. Since 1980 in European countries, improvements in quality of medical care processes have been linked to gains in life expectancy (Nolte et al., 2002). A joint study from Boston, Harvard and Stanford Universities noticed that areas with a larger number of primary care physicians registered lower mortality rates in the United States (Basu et al., 2019). Adding 10 family doctors per 100,000 people decreased mortality; an increase of 10 primary care physicians per 100,000 population was associated with approximately 52 more days of life expectancy, whereas an additional 10 specialist doctors were related to 19 more days.

Despite this positive correlation between physicians and life expectancy, the number of primary doctors has decreased in several OECD countries (OECD, 2008). Possible reasons for this were lower pay and prestige (Vogel, 2019) or cases where the supply of primary care doctors is regulated by the market. Physician remuneration inequalities were documented in the UK in the 1995–2004 period, but problems were also attributed to expatriates, causing a shortage of doctors in their home country (Tjadens et al., 2013). Based on the above-mentioned considerations, we posit the following hypothesis:

#### HP3

*Greater supply of medical care components (physician and hospital beds) is positively associated with life expectancy.*

### Health habits and population health

Dietary determinants and life risk factors like smoking, alcohol consumption, and sugar and fat intake were commonly addressed as important variables to measure longevity in different countries (Cochrane et al., 1978; Laranjeira & Szrek, 2016; Nixon & Ulmann, 2006; Park & Nam, 2018).

Prolonged tobacco use can cause several diseases such as vascular, prostate, lung, and breast cancer (Lariscy, 2019); cutting tobacco consumption by two cigarettes a day can increase life expectancy (Shaw et al., 2005). In developed countries, during the 1990s, tobacco was responsible for about 30% of all deaths between the ages of 35 and 69 and for 14% of deaths for older individuals, showing that between 1950 and 2006 smoking played a major role in determining population mortality (Peto et al., 1992).

Alcohol consumption too has a negative impact on longevity as it contributes to cancer of the mouth or oesophagus, ischaemic stroke, and diabetes mellitus. Heavy alcohol consumption affects employment opportunities, and, at the same time, prolonged unemployment may lead to higher risk of alcohol intake (Anderson & Baumberg, 2006). Additional studies showed that when moderate drinking was combined with smoking, the risk of disease, especially cancer, increased and shorter life expectancies were observed (Xu et al., 2007). Similarly, poor diets with a high percentage of fat and sugar intake had negative effects on life expectancy with premature mortality (Barlow & Vissandjée, 1999; Berger & Messer, 2002).

Previous studies have also found a negative relationship between chronic diseases and life expectancy, with life expectancy decreasing by 1.8 years with each additional chronic condition (Dugoff et al., 2014). Chronic respiratory diseases, including chronic obstructive pulmonary disease, asthma, occupational lung diseases and pulmonary hypertension, are an important contributor to the slowing life expectancy improvements and seem to negatively affect life expectancy (GBD Chronic Respiratory Disease Collaborators, 2020; Shavelle et al., 2009). Based on this, we propose this hypothesis:

#### HP4

*Health habits like alcohol consumption and population health such as chronic respiratory diseases are negatively correlated to life expectancy.*

### Social determinants

Social determinants of health are defined by the WHO as “the conditions in which people are born, grow, live, work and age”. Socioeconomic factors were considered to be as relevant as medical care in determining health outcomes (Braveman & Gottlieb, 2014; Braveman et al., 2011; Exworthy, 2008). Disparities in health according to income were similar between countries with different access to health care as in the UK and the USA (Martinson, 2012). Social inequalities represent a “fundamental cause of health” (Kaplan & Keil, 1993) because they can influence multiple diseases and risk factors. Findings pointed out that higher education levels generally lead to much healthier behaviour, less exposure to life-threatening factors (i.e., smoking, alcohol consumption, poor diet); and better working conditions allow broader access to health insurance options, whereas social position brings more economic resources (Avendano et al., 2009; Braveman et al., 2011).

Employment and labour force rate were also often considered to be social determinants of life expectancy (Rogot et al., 1992). Health is an essential element of human capital: better health increases participation in the labour market and productivity. A deterioration in health behaviours was observed during long periods of unemployment (Janlert et al., 2014). Since the 2008 recession, new studies, especially from the Anglo–Saxon and Nordic countries, have focused on the positive impact of active labour market policies on longevity (Puig-Barrachina et al., 2020). Based on these issues, we propose the following hypothesis:

#### HP5

*Social determinants of health and particularly the share of population that is economically active are associated with life expectancy.*

### Social spending

Social spending and social protection expenditures, often taken as a share of the GDP, are ways in which countries assume responsibility for supporting disadvantaged or vulnerable groups of people.

Social expenditure consists of benefits (both in cash and in kind) and tax waives for social purposes. Benefits may involve low-income households, the elderly, disabled, sick, unemployed, young and homeless. The different ways of providing public benefits have a significant impact on household disposable income and on consumption. In the Euro area, increases in social goods and services paid directly in cash increase household gross disposable income that can be utilised for commodities or savings, subsequently improving the chances to achieve a higher standard of living and health. In countries where a high percentage of the GDP is spent on social protection, fewer unmet health care needs were reported and the population had a significant longer life expectancy (Heuvel & Olaroiu, 2017). Although prior studies have underlined the tendency for Europeans to be generally healthier than Americans despite less spending on health care (Anderson & Frogner, 2008; Avendano et al., 2009), some findings suggest that population health depends not only on universal access to health care but on the level of investments in social policies and social programmes (Elola et al., 1995; Lobb, 2009). Based on previous research, we formulate this hypothesis:

#### HP6

*Social protection expenses, measured by social spending over GDP, are positively correlated with population life expectancy.*

### Other external factors

External factors are a residual category mainly related to hygienic conditions, the environment, the economic and social context, and innovation. Sanitation was found to be an important factor for population health and longevity (Ranabhat et al., 2018). In OECD countries, access to safe sanitation prevented more than 700,000 deaths each year.

Air pollution is also noxious to population health. Ozone, nitrogen oxides, PM 2.5, PM 10 and sulphur oxides were often considered to determine the impact of air pollution on life expectancy. Their reductions have been shown to improve public health benefits (Brunekreef & Holgate, 2002; Hill et al., 2019). Pollution seemed to increase in the presence of income inequalities (Hill et al., 2019; Naryan & Naryan, 2008). According to several studies, even air temperature can affect longevity and it is an important determinant of health (Gasparrini et al., 2015; Odhiambo Sewe et al., 2018). One’s economic and social context are external factors related to the GDP and population levels. The GDP, both in absolute terms and per capita, influences life expectancy, meaning that income has an effect on health indicators (Blázquez-Fernández et al., 2018). Swift (Swift, 2011) observed in 13 OECD countries during the period 1820–2001 a relationship between life expectancy (longevity) and GDP. A 1% increase in life expectancy resulted in an average 6% increase in GDP. Regarding the population size, the literature revealed non-conclusive results: if on the one hand a high reproductive timing delay has negative effects on longevity (Bulled & Sosis, 2010; Goldstein & Schlag, 1999), on the other hand, an increase in population density could have either positive or negative effects (Cochrane et al., 1978; Crémieux et al., 1999). Health policy also plays a crucial role in life expectancy by improving quality of care and by promoting a healthy lifestyle. Maximising population health outcomes and reducing health inequities form the basis of the Sustainable Development Goals of the United Nations 2030 Agenda for Sustainable Development. National and international reviews have identified general areas for policy action to target the social determinants of health and health inequities within the WHO European Region.

Innovation in health care could be another factor in determining longevity. Pharmaceutical advancements have made important contributions to ameliorating functional limitations of older people (Crémieux et al., 2005; Lichtenberg, 2017), whereas improvements in medical care protocols and machine technologies reduced mortality and morbidity (Laranjeira & Szrek, 2016). Considering the previous analysis, we posit the following research hypothesis:

#### HP7

*Other external variables, such as GDP levels, population size and air temperature, are associated with life expectancy.*

## Data and model specification

Our purpose was to study the relationships between life expectancy and a set of independent variables with a “holistic” approach in a single model, considering all seven categories that we identified in our literature review and selecting at least one variable from each. In choosing the variables, one constraint was the availability of data from reliable public sources for the years 1999–2018 and for all the OECD countries. Table 1 contains a definition of all the variables considered in our model and their source. We mostly used data from the World Bank, from the World Health Organization, and from OECD databases and only in a few cases from the Institute for Health Metrics and Evaluation (University of Washington), and the International Labour Organization. We took the original measures available from the data sources, with the exception of two variables (GDP_Q1 and GDP_Q5) that we converted in dummies.

**Table 1:** Variable definition

Variable	Definition	Source
LIF_EXP_T	Life expectancy at birth, overall in years	WB1
*Health care expenditures*		
H_EXP_T	Logarithm of total health expenditures per capita (PPP dollars, deflated)	WHO1
*Health financing policies*		
H_EXP_O	Share of total out–of–pocket payments to total current health expenditure	WHO1
*Elements of medical care*		
PHYSIC	Number of physicians per 1,000 inhabitants	WHO2
HOS_BED	Number of hospital beds per 1,000 inhabitants	WHO2
*Health habits and population health*		
CAL	Logarithm of kilocalories per capita per day	OECD1
ALC	Alcohol liters per capita per year	IHME
RESP	Prevalence (share) of respiratory diseases	IHME
*Social determinants*		
LAB_FOR	Share of population aged 15 or more that is economically active	ILO
*Social spending*		
SOC	Share of public social spending to GDP	OECD2
*Other external factors*		
GDP_Q1	Dummy = 1 if in the lowest 20% of per capita GDP	WB1
GDP_Q5	Dummy = 1 if in the highest 20% of per capita GDP	WB1
TEMP	Monthly average temperature (Celsius degrees)	WB2
POPUL	Logarithm of population size	WB1

The data sources are; *IHME*: Institute for Health Metrics and Evaluation, Global Health Data Exchange (https://ghdx.healthdata.org/); *ILO*: International Labour Organization, ILOSTAT database (https://ilostat.ilo.org/data/#); *OECD1*: Organization for Economic Cooperation and Development, OECD STAT, Non-medical determinants of health (https://stats.oecd.org/); *OECD2*: Organization for Economic Cooperation and Development, Social spending (indicator). (https://data.oecd.org/socialexp/social-spending.htm); *WB1*: The World Bank, DataBank (https://data.worldbank.org/indicator/); *WB2*: The World Bank, Climate Knowledge portal (https://climateknowledgeportal.worldbank.org/); *WHO1*: World Health Organization, Global Health Expenditure Database (https://apps.who.int/nha/database); *WHO2*: World Health Organization, Health Care Resources (https://stats.oecd.org/)

The dependent variable is life expectancy (LIFE_EXP_T), defined as the average number of years a person can expect to live from birth, supposing constant age-specific mortality rates. We selected the following explanatory variables to be included in the seven categories. For the first one, *health care expenditures*, we chose the total per capita health care expenditure (H_EXP_T). Regarding *health financing policies*, we used the out-of-pocket incidence (over total health expenditure, H_EXP_O). We did not consider universal coverage because almost all OECD countries offer universal coverage funded by national funds or private entities. Considering *elements of medical care*, we chose two variables: the number of physicians (PHYSIC) and hospital beds (HOS_BED) per 1000 inhabitants. Regarding *health habits and population health*, we considered citizen behaviours such as total kilocalorie intake per capita per day (CAL), alcohol consumption (ALC), and the prevalence of chronic respiratory diseases (RESP). Concerning *social determinants*, we used the labour force participation rate (LAB_FOR), whereas regarding *social spending* we considered the public social spending as a share of GDP (SOC). For *other external factors*, we created two dummy variables (GDP_Q1 and GDP_Q5), which refer to the first and last quintile of (per capita) GDP in our sample, the average monthly atmospheric temperature (TEMP), and the size of the population (POPUL).

Table 2 contains summary statistics for the aforementioned variables, including mean, standard deviation, minimum and maximum for up to 20 years (from 1999 to 2018) on 36 OECD countries.^2^ The total number of observations is 601, with an average 16.69 observations per country (ranging from 8 for Japan to 19 for ten countries). The number of observations per country is lower than 20 because of missing values in some variables for certain years.

**Table 2:** Summary statistics (601 observations)

Variable name	Mean	Std. dev	Min	Max
LIF_EXP_T	78.797	3.095	70.259	83.602
H_EXP_T	7.951	0.551	6.675	9.188
H_EXP_O	0.212	0.095	0.071	0.557
PHYSIC	3.138	0.795	0.947	6.353
HOS_BED	5.032	2.248	1	14.690
CAL	8.117	0.072	7.895	8.265
ALC	9.527	2.782	1.200	14.800
RESP	0.102	0.033	0.039	0.176
LAB_FOR	0.603	0.062	0.455	0.838
SOC	0.197	0.058	0.044	0.322
GDP_Q1	0.201	0.401	0	1
GDP_Q5	0.200	0.400	0	1
TEMP	9.812	5.160	–7.100	22.700
POPUL	16.329	1.467	12.649	19.593

Life expectancy has a mean value of 78.80 years with a range of about thirteen years (from 70.26 to 83.60 years). Country expenses for health (both private and public) is on average exp(7.951) = 2838 USD, varying from less than 1000 USD per capita (like in Colombia, Latvia and Poland) to about 10,000 USD per capita (the United States). Out-of-pocket expenses account on average for 21.2% of total health expenses and vary greatly from 7.1 to 55.7%. Physicians per 1000 inhabitants range from about 1 to more than 6 (average around 3), whereas the other medical care element considered, hospitals beds, has an even greater dispersion (the mean is about 5, ranging from 1 to 15). Workforce (people aged 15 or more that are economically active) is on average 60.3%, with a moderate standard deviation and maximum peak of 83.8%. Social spending (which considers all public expenses related to social spending) accounts for 19.7% of GDP and varies from 4.4 to 32.2%. Regarding individual behaviours, daily calorie intake is about exp(8.117) = 3350 (ranging from 2700 to 3900), whereas annual alcohol consumption (litres) varies from 1.2 to 14.8, a mean of 9.53. Chronic respiratory diseases have a share of 10.2% in the population, varying from 3.9 to 17.6%. The average annual atmospheric temperature is 9.81 degrees Celsius, ranging from an average of − 7.1 to 22.7.

### Methodology

We ran fixed-effect OLS regressions, where the dependent variable is life expectancy at birth (measured in years) and the specification includes all the variables summarized in Table 1. Specifically, we consider the following benchmark equation for country *i* in year *t*:

1 $$LE_{it} = \beta_{0} + HC_{it}^{^{\prime}} \beta_{1} + \beta_{2} HF_{it} + MC_{it}^{^{\prime}} \beta_{3} + HP_{it}^{^{\prime}} \beta_{4} + \beta_{5} SD_{it} + \beta_{6} SS_{it} + O_{it}^{^{\prime}} \beta_{7} + Y_{t} + C_{i} + \varepsilon_{it}$$

where the $\beta s$ are the coefficients to be estimated, $\varepsilon$ is the idiosyncratic error term, *Y* are year fixed effects and *C* country fixed effects. The dependent variable in Eq. (1) is life expectancy (*LE*), which is regressed over a specification including the seven categories of potential determinants: health care expenditures (*HC*); health financing policies (*HF*); elements of medical care (*MC*); health habits and population health (*HP*); social determinants (*SD*); social spending (*SS*); and other external factors (*O*). Four out of the seven categories (*HC*, *MC*, *HP* and *O*) include more than one variable.

Table 3 reports the pairwise correlation matrix among the variables considered. Life expectancy shows the highest pairwise correlation with total health expenditures (0.75). Most explanatory variables are cross-correlated, but the correlation is usually small.

**Table 3:** Pairwise correlation matrix (only significant correlations)

	(1)	(2)	(3)	(4)	(5)	(6)	(7)	(8)	(9)	(10)	(11)	(12)	(13)	(14)
(1) LIF_EXP_T	1													
(2) H_EXP_T	0.754	1												
(3) H_EXP_O	− 0.401	− 0.612	1											
(4) PHYSIC	0.256	0.268	− 0.087	1										
(5) HOS_BED	− 0.196	− 0.135		0.126	1									
(6) CAL	0.296	0.486	− 0.271	0.256	− 0.130	1								
(7) ALC	− 0.113	0.120	− 0.264	0.297	0.470		1							
(8) RESP	0.565	0.737	− 0.508	0.160	− 0.243	0.421	0.141	1						
(9) LAB_FOR	0.349	0.428	− 0.224		− 0.210		− 0.083	0.323	1					
(10) SOC	0.432	0.523	− 0.541	0.485	0.152	0.324	0.350	0.505	− 0.157	1				
(11) GDP_Q1	− 0.684	− 0.689	0.497	− 0.245		− 0.368		− 0.498	− 0.279	− 0.401	1			
(12) GDP_Q5	0.350	0.628	− 0.309	0.122	− 0.157	0.294		0.374	0.474	0.084	− 0.251	1		
(13) TEMP		− 0.259	0.325		− 0.143		− 0.169		− 0.366	− 0.161	0.104	− 0.289	1	
(14) POPUL	0.105			− 0.312		0.132	− 0.237	0.083	− 0.285			− 0.181	0.298	1

601 observations. Only correlations that are significant at least at the 5% level are reported

**Fig. 1:**
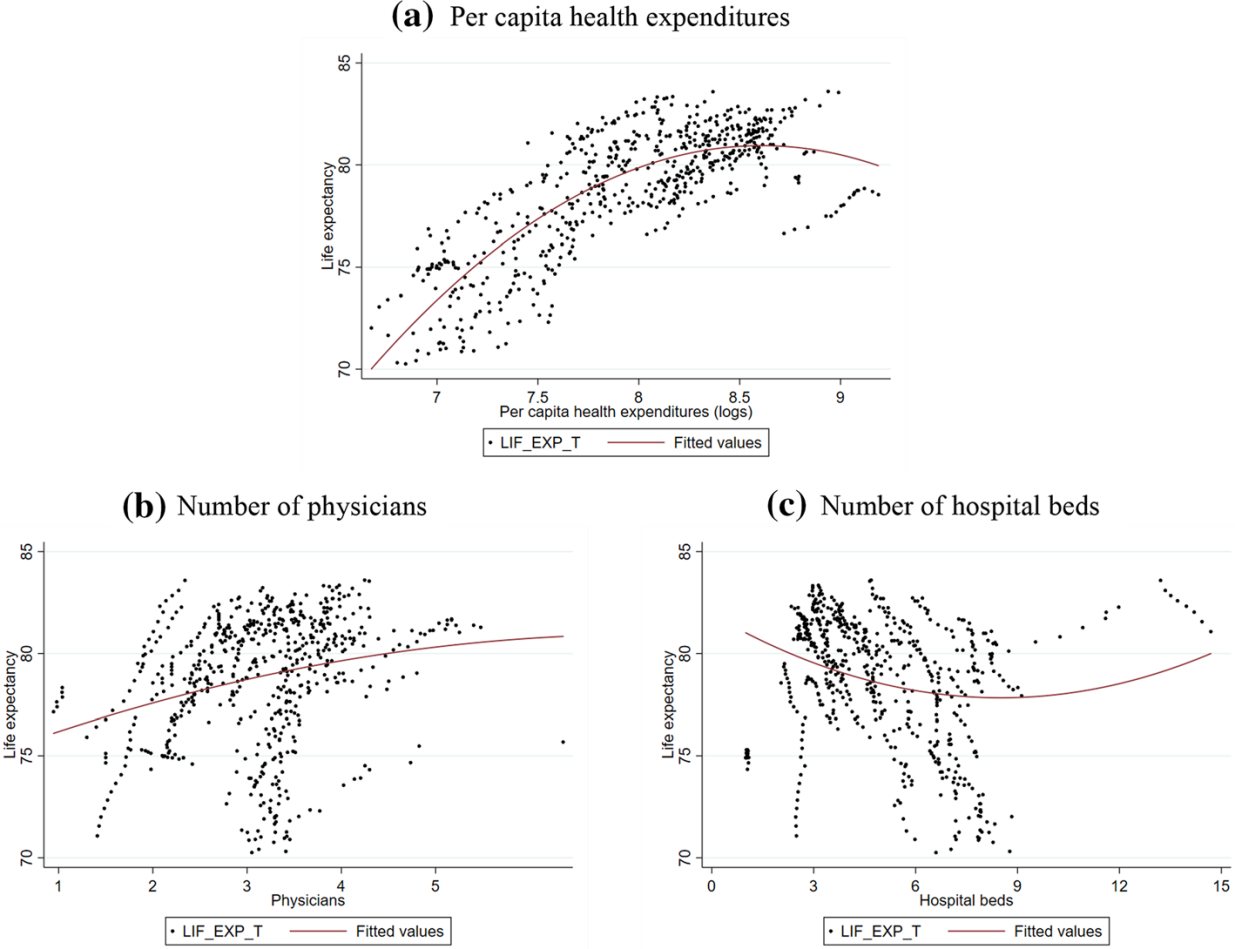
Observed trajectories of life expectancy

Three explanatory variables (total health expenditures, kilocalories per day and population size) are used in logarithms to reduce their variability and simplify the interpretation of the coefficients so that they can be read as semi-elasticities. In addition, in the specification we include squared powers of total health expenditures, number of physicians and number of hospital beds, because the data seem to suggest a non-linear pattern (see Fig. 1).

Fixed-effect OLS models correct for endogeneity deriving from time-invariant omitted variables, which is absorbed in the country fixed effects. Statistical tests confirm that we should prefer a fixed-effect model to a pooled model (Chow test: 150.02; p value < 0.001) and to a random-effect model (Hausman test: 180.79; p value < 0.001). Moreover, we found no evidence of quasi-collinearity among the explanatory variables (the VIF ranged from 1.46 to 8.06).

However, endogeneity could still be arise in the form of reverse causality, meaning that life expectancy has an impact on some same-year explanatory variables such as total expenditures or respiratory diseases. For this reason, in a robustness check of the analysis we estimated two alternative models. In one case we considered dynamic—rather than static—fixed-effect models in which the explanatory variables are observed some years before the dependent variable. For instance, if the explanatory variables are observed one year before the dependent variable, we estimate Eq. (2):

2 $$LE_{it} = \beta_{0} + HC_{it - 1}^{^{\prime}} \beta_{1} + \beta_{2} HF_{it - 1} + MC_{it - 1}^{^{\prime}} \beta_{3} + HP_{it - 1}^{^{\prime}} \beta_{4} + \beta_{5} SD_{it - 1} + \beta_{6} SS_{it - 1} + O_{it - 1}^{^{\prime}} \beta_{7} + Y_{t} + C_{i} + \varepsilon_{it}$$

In another case we implemented the Arellano–Bond estimator. This estimator is essentially a fixed-effect regression applied to Eq. (1), where the explanatory variables (i.e., all the variables belonging to the seven categories of potential determinants of life expectancy) are instrumented using their most recent lags (up to five). Both approaches should remove the problem of reverse causality, as life expectancy cannot have implications on variables that originated earlier.

## Results

This section presents the results of our analysis, which are reported in Tables 4 and 5. We have chosen to comment only on coefficients that are significant at least at the 5 percent level. The bottom of each table reports the p-value of three separate F-tests on the joint significance of the coefficients involving a quadratic term: health expenditure (variables H_EXP_T and (H_EXP_T)^2^); physicians (variables PHYSIC and (PHYSIC)^2^); and hospital beds (variables HOS_BED and (HOS_BED)^2^). In most cases the tests usually report a significant association with life expectancy.

**Table 4:** Benchmark results

	(1)	(2)	(3)	(4)	(5)
Timing of the regressors	Contemporaneous	1–year lag	3–year lag	5–year lag	Contemporaneous
Model	Fixed-effect	Fixed-effect	Fixed-effect	Fixed-effect	Arellano-Bond
H_EXP_T	5.879***	4.537**	4.294*	7.964***	5.340***
	(2.050)	(2.139)	(2.217)	(2.136)	(0.890)
(H_EXP_T)^2^	− 0.385***	− 0.296**	− 0.281**	− 0.494***	− 0.338***
	(0.131)	(0.137)	(0.142)	(0.138)	(0.057)
H_EXP_O	3.981***	3.862***	2.819**	1.424	4.202***
	(1.042)	(1.087)	(1.092)	(1.049)	(0.444)
PHYSIC	0.458*	0.618**	0.319	0.059	0.492***
	(0.240)	(0.275)	(0.288)	(0.301)	(0.103)
(PHYSIC)^2^	− 0.075**	− 0.112***	− 0.071	− 0.018	− 0.077***
	(0.032)	(0.040)	(0.043)	(0.047)	(0.014)
HOS_BED	− 0.339***	− 0.157	− 0.264	− 0.406**	− 0.005
	(0.124)	(0.147)	(0.162)	(0.175)	(0.060)
(HOS_BED)^2^	0.035***	0.023**	0.030**	0.036**	0.012***
	(0.009)	(0.010)	(0.012)	(0.014)	(0.004)
CAL	0.464	0.238	4.066***	4.390***	0.753
	(1.044)	(1.115)	(1.208)	(1.181)	(0.463)
ALC	− 0.142***	− 0.095***	− 0.036	0.024	− 0.149***
	(0.030)	(0.031)	(0.032)	(0.030)	(0.013)
RESP	− 11.307***	− 15.439***	− 10.157**	− 4.250	− 20.342***
	(3.588)	(3.973)	(4.256)	(4.251)	(1.845)
LAB_FOR	6.052***	5.666***	5.580***	4.772***	6.329***
	(1.208)	(1.261)	(1.386)	(1.520)	(0.533)
SOC	6.161***	7.960***	8.481***	5.461***	8.060***
	(1.612)	(1.668)	(1.690)	(1.598)	(0.718)
GDP_Q1	− 0.308***	− 0.390***	− 0.376***	− 0.333***	− 0.326***
	(0.094)	(0.095)	(0.093)	(0.088)	(0.040)
GDP_Q5	0.016	0.057	0.014	− 0.039	− 0.144***
	(0.106)	(0.115)	(0.121)	(0.117)	(0.048)
TEMP	− 0.001	0.010	0.083**	0.055	0.007
	(0.039)	(0.042)	(0.041)	(0.038)	(0.017)
POPUL	− 3.004***	− 3.686***	− 4.387***	− 4.754***	− 2.328***
	(0.655)	(0.692)	(0.757)	(0.845)	(0.282)
Constant	96.382***	113.548***	94.453***	84.239***	
	(18.089)	(19.139)	(19.896)	(20.866)	
Year effects	YES	YES	YES	YES	YES
F-Test health expenditures = 0	[0.013]	[0.098]	[0.141]	[0.001]	[0.000]
F-Test physicians = 0	[0.044]	[0.005]	[0.064]	[0.770]	[0.000]
F-Test hospital beds = 0	[0.000]	[0.000]	[0.000]	[0.032]	[0.000]
Within-group R-squared	0.909	0.906	0.900	0.902	
Mean VIF	2.53	2.52	2.60	2.61	2.53
Countries	36	35	35	35	35
Observations	601	550	481	415	550

The dependent variable is LIF_EXP_T. The model is fixed-effect OLS in Columns (1)–(4) and fixed-effect Arellano–Bond in Column (5). Standard errors in round parentheses; p–values in squared parentheses. ***p < 0.01, **p < 0.05, *p < 0.1

**Table 5:** Robustness checks: Specification and sample

	(1)	(2)	(3)	(4)
	Stepwise selection	EU countries	Universal coverage	Mixed coverage
H_EXP_T	5.827***	9.657***	10.634***	5.566**
	(1.921)	(2.278)	(2.333)	(2.417)
(H_EXP_T)^2^	− 0.380***	− 0.619***	− 0.686***	− 0.369**
	(0.122)	(0.147)	(0.151)	(0.156)
H_EXP_O	3.931***	− 1.556	− 1.174	4.711***
	(1.033)	(1.155)	(1.152)	(1.129)
PHYSIC	0.461*	1.683***	1.303***	0.627**
	(0.238)	(0.359)	(0.334)	(0.274)
(PHYSIC)^2^	− 0.075**	− 0.200***	− 0.162***	− 0.095***
	(0.032)	(0.044)	(0.043)	(0.036)
HOS_BED	− 0.348***	0.167	− 0.269**	− 0.270**
	(0.121)	(0.189)	(0.130)	(0.134)
(HOS_BED)^2^	0.036***	− 0.012	0.025***	0.030***
	(0.008)	(0.016)	(0.009)	(0.009)
CAL		2.071*	2.197**	1.446
		(1.161)	(1.102)	(1.164)
ALC	− 0.140***	− 0.238***	− 0.227***	− 0.146***
	(0.029)	(0.031)	(0.031)	(0.033)
RESP	− 11.547***	− 15.029***	− 3.801	− 11.207**
	(3.536)	(4.642)	(4.472)	(4.600)
LAB_FOR	6.065***	3.697***	3.923***	6.432***
	(1.197)	(1.201)	(1.221)	(1.328)
SOC	5.956***	4.713***	4.230**	8.479***
	(1.524)	(1.646)	(1.802)	(1.886)
GDP_Q1	− 0.305***	− 0.397***	− 0.326***	− 0.301***
	(0.093)	(0.096)	(0.097)	(0.099)
GDP_Q5		− 0.054	0.008	− 0.056
		(0.110)	(0.133)	(0.126)
TEMP		− 0.049	− 0.040	− 0.004
		(0.048)	(0.042)	(0.045)
POPUL	− 3.057***	− 1.923**	− 3.047***	− 3.394***
	(0.644)	(0.767)	(0.751)	(0.763)
Constant	111.221***	50.905**	65.000***	94.560***
	(16.801)	(20.096)	(19.602)	(20.220)
Year effects	YES	YES	YES	YES
F-Test health expenditures = 0	[0.006]	[0.000]	[0.000]	[0.056]
F-Test physicians = 0	[0.040]	[0.000]	[0.001]	[0.026]
F-Test hospital beds = 0	[0.000]	[0.633]	[0.001]	[0.000]
Within-group R squared	0.909	0.934	0.932	0.906
Mean VIF	2.30	3.00	2.79	2.68
Countries	36	25	27	32
Observations	601	441	450	532

The dependent variable is LIF_EXP_T; all the explanatory variables are contemporaneous. The model is fixed-effect OLS. Column (1) includes all the countries in the dataset; Column (2) includes the following countries: Austria, Belgium, Czech Republic, Denmark, Estonia, Finland, France, Germany, Greece, Hungary, Iceland, Ireland, Italy, Latvia, Lithuania, Luxembourg, Netherlands, Norway, Poland, Portugal, Slovak Republic, Slovenia, Spain, Sweden, and United Kingdom. Column (3) includes the following countries: Australia, Belgium, Canada, Czech Republic, Denmark, Estonia, Finland, France, Greece, Hungary, Iceland, Ireland, Italy, Japan, Korea, Latvia, Lithuania, Luxembourg, New Zealand, Norway, Poland, Portugal, Slovak Republic, Slovenia, Spain, Sweden, and United Kingdom. Column (4) includes the following countries: Australia, Austria, Belgium, Canada, Chile, Czech Republic, Denmark, Estonia, Finland, France, Germany, Greece, Hungary, Iceland, Ireland, Italy, Japan, Korea, Latvia, Lithuania, uxembourg, Mexico, New Zealand, Norway, Poland, Portugal, Slovak Republic, Slovenia, Spain, Sweden, Turkey, and United Kingdom. Standard errors in round parentheses; p–values in squared parentheses. ***p < 0.01, **p < 0.05, *p < 0.1

Table 4 shows our benchmark results. Column (1) considers a static model in which dependent and explanatory variables are observed in the same year. The fit of the model was generally high (within-group R-squared: 0.91), which suggests that this specification is able to capture a large part of the variability in the data. Many variables were correlated with life expectancy. Health expenditures revealed a quadratic link to life expectancy, which initially increased with health expenditures up to a maximum expenditure that the model sets at 2069.51 USD (exp(5.879/(2*0.385))), below the average in the sample (which is exp(7.951) = 2838 USD). Several studies found a positive association of this determinant (Crémieux et al., 2005; Jaba et al., 2014; Nixon & Ulmann, 2006; Park & Nam, 2018). In our study the relationship was initially positive but once the maximum amount was reached, any further increase in expenditures had a negative relationship with life expectancy.

We found that the ratio of out-of-pocket to total expenditures is positively associated with health expectancy. Grima et al. (Grima et al., 2018) found that out-of-pocket payments positively influence life expectancy at birth, whereas Moreno-Serra and Smith (Moreno-Serra & Smith, 2014) found that out-of-pocket health spending as a share of the total health expenditure is linked to lower adult mortality.

The number of physicians has an inverted U-shaped association with an initial positive correlation with life expectancy, but only when the physicians are up to 3.05 [0.458/(2*0.075] per 1000 inhabitants (in line with the average in the sample, which is 3.14). Previous studies found a positive correlation (Cochrane et al., 1978; Crémieux et al., 1999; Nixon & Ulmann, 2006; Vogel, 2019), confirming the relevance of these components of the health care system, which provide both general and hospital care. Regarding hospital beds, we found a U-shaped (non-inverted) correlation with life expectancy, which after an initial fall increased with the number of beds, but only when there are at least 4.84 (0.339/(2*0.035)) beds per 1000 inhabitants. In the Babazono and Hilman (Babazono & Hillman, 1994) study, the number of beds was found to be significant in decreasing mortality rates whereas, according to Rhee (Rhee, 2012), life expectancy is immediately affected by health-related facilities in the short run, but less in the long run.

Focusing on alcohol consumption and chronic respiratory diseases, we found significant negative relationships with life expectancy, in line with previous studies (Berger & Messer, 2002; Crémieux et al., 1999, 2005). In particular, one more alcohol litre per capita per year is associated with a 0.142 year reduction in life expectancy, while a 10 percentage point increase in the prevalence of respiratory diseases is associated with a 1.1307 year decrease in life expectancy. As also observed by Berger and Messer (Berger & Messer, 2002) and Rogot et al. (Rogot et al., 1992), labour force participation had a positive correlation with life expectancy, similarly to social spending (Heuvel & Olaroiu, 2017); a 10 percentage point increase in labour force (social spending) is associated with a 0.605 (0.616) year increase in life expectancy. Low GDP per capita is associated with reduced life expectancy, in line with Blázquez–Fernandez et al. (Blázquez-Fernández et al., 2018) and Swift (Swift, 2011). Countries in the first quintile (Q1) of GDP showed a statistically lower life expectancy (by 0.308 years). Conversely, countries in the last quintile showed a non-significant relationship, probably due to the health care outcomes in countries such as the United States, where high rates of health care spending are not related to long life expectancy. In a similar fashion we did not find significant associations for the daily calorie intake and air temperature, in contrast with some previous evidence (e.g., Aisa et al. (Aisa et al., 2014); Bunker et al. (Odhiambo Sewe et al., 2018)). Lastly, we found that the (log of) population size has a negative correlation with life expectancy (a 10% increase in population size is associated with a 0.30 year reduction in life expectancy), in line with Cervellati and Sunde (Cervellati & Sunde, 2011) and Crémieux et al. (Crémieux et al., 1999). It is possible that, the more populated a country, the more difficult it is to access health care or social services.

**Fig. 2:**
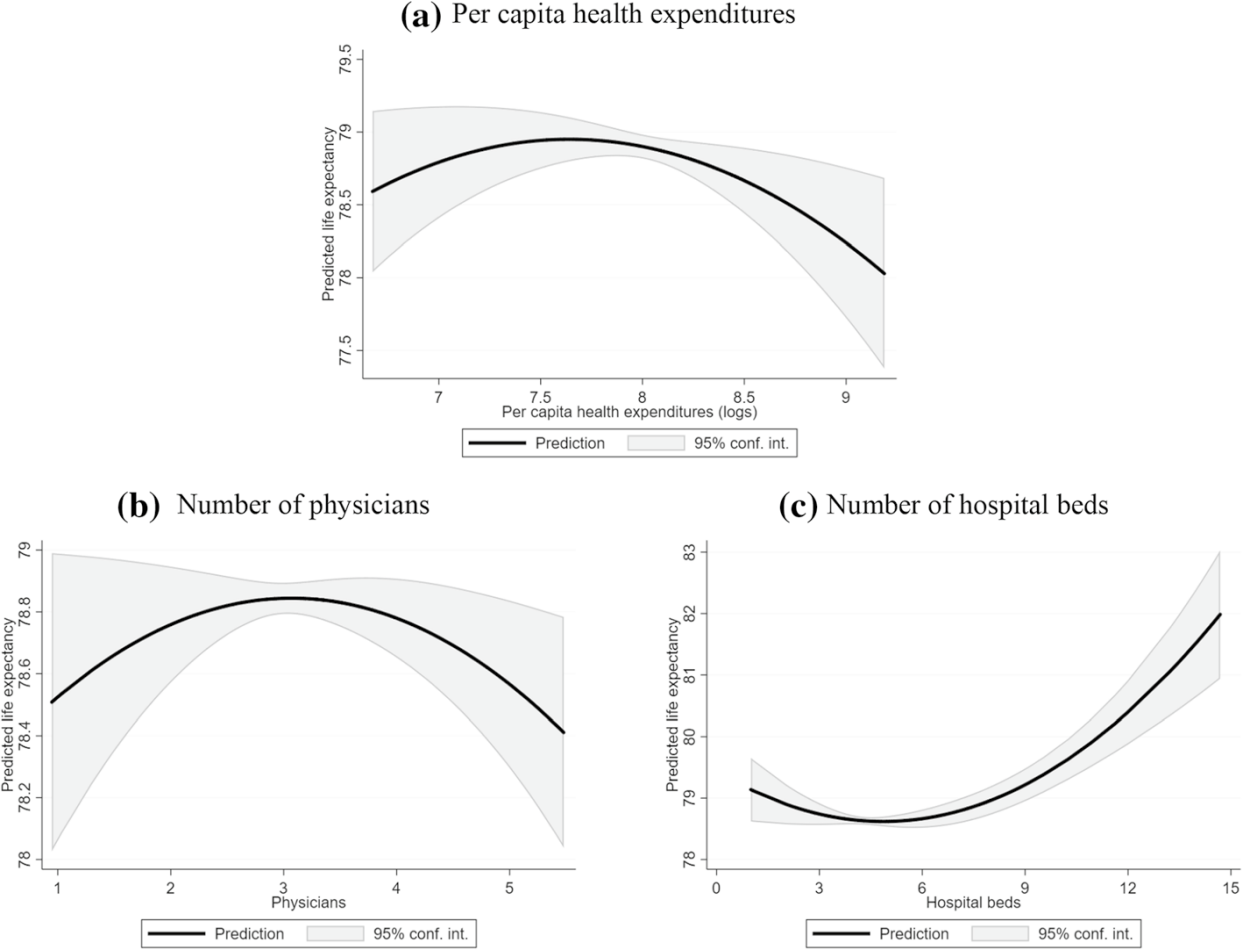
Predicted trajectories of life expectancy. (*Note*: Predictions are based on the output from the regression model in Table 4, Column (1). All the other explanatory variables are kept at their average)

Figure 2 reports the predicted trajectories of life expectancy based on the values of total health expenditures, the number of physicians and the number of hospital beds. Predictions are based on the model in Column (1) of Table 4, keeping the other explanatory variables fixed at their average. Although the trend is clear, we noticed only small variations in the predicted life expectancy, which in most cases ranges between 78 and 79.5 years. The exception is the profile for the number of hospital beds that gives rise to predictions of life expectancy over 80 years, starting from 9 beds per 1000 inhabitants. This evidence could be driven by some outliers (see Fig. 1, panel c), which mainly regard Japan.

Excluding Japan, or using a cubic polynomial on the number of hospital beds, we obtained similar results (available upon request). In our opinion, this indicates that the number of hospital beds has a crucial relationship with life expectancy.

Repeating the regression analysis using standardised variables (that is, variables that are transformed in such a way to have mean 0 and standard deviation (1), the coefficients measure the effect on life expectancy of a relative change by one standard deviation in the explanatory variables. The output, shown in Appendix Table 6, informs that the two most powerful explanatory variables in the model of Column (1) are population size and total (per capita) health care spending, which highlights their important contribution in the relationship with life expectancy. Policy makers could carefully consider these results in defining and allocating public spending, particularly after the COVID-19 pandemic period that should drive countries to new investments in their HCS.

### Robustness checks

In this sub-section we report the output from robustness checks along three dimensions: (i) the static/dynamic nature of the specification, (ii) the number of explanatory variables and (iii) the sample composition.

Regarding i), in Columns (2)-(4) of Table 4 we present dynamic models in which the explanatory variables are observed some years before the dependent variable. There is no general rule informing on an adequate year delay to consider, and for this reason in Table 4 we considered a one-year lag (Column 2), a three-year lag (Column 3) and a five-year lag (Column 4). This means that, if life expectancy is observed in year *t*, the explanatory variables are observed in years *t-1*, *t-3*, and *t-5* respectively. Of course, the higher the lag, the smaller the sample size. Our benchmark results were generally preserved, both qualitatively and quantitatively. In the new regressions we occasionally found additional significant effects of calorie intake (positively, in Columns 3 and 4) and air temperature (positively, in Column 3). Table 4 concludes by reporting in Column (5) the output from an Arellano–Bond estimator, where the explanatory variables are instrumented using their most recent lagged values (up to five). All our key findings are confirmed.

In the second type of robustness check, shown in Column (1) of Table 5, we adopted a backward stepwise selection to keep in the final specification only the explanatory variables that contribute to describe the dependent one. This approach is purely statistical, but helps to highlight whether the excellent fit of the model in Table 4 depends on the inclusion of a relatively large number of explanatory variables, and if some of the effects we commented are spurious and possibly due to the inclusion of superfluous variables. The output suggests that this is not the case, as only three variables were removed from the specification (calorie intake, high GDP, and air temperature), whereas all the other variables were kept and exhibited coefficients with the same sign and similar size as the model in Column (1) of Table 4. Moreover, the fit was the same (0.91) as in Column (1) of Table 4 (reference model).^3^

The third and final type of robustness check is also shown in Table 5. It replicated the model of Table 4, Column (1) in a reduced sample that includes only EU countries (Column 2), or countries with specific health systems. In particular, we looked at countries with universal public health systems (Column 3), and universal public or mixed public–private health systems (Column 4). Depending on the sample, from time to time we lost significance in some coefficients. Those that always remained significant were heath expenditure, the number of physicians, alcohol intake, labour force participation, social spending, low GDP and population size. Interestingly, the two coefficients of health expenditures were no longer (marginally) significant in the model of Column (4) jointly considered (see F-test at the bottom of the table). The general picture we obtained from the benchmark analysis is nevertheless confirmed.

## Conclusion, limitations and future research

Life expectancy at birth is one of the most important variables to use for a global evaluation of a country’s well-being. Previous studies, considering single countries or limited groups of countries, already pointed out the relationship among health care expenditure, social or economic behaviours and life expectancy. Unfortunately, these analyses often used only a few variables at a time and for short periods; they did not use recent data or consider many countries. Our study, based on a dataset covering 20 years (1999–2018), fills this gap, focuses on 36 OECD countries and proposes a model to jointly evaluate the relationship between many explanatory variables and life expectancy at birth.

We formulated seven hypotheses associated with the groups of variables that we identified from the economic literature. To test these hypotheses, we ran multiple regression analyses and made robustness checks using dynamic models with lagged explanatory variables for one, three or five years, stepwise backward selection and an Arellano Bond estimator. Results were substantially stable and largely confirmed our hypotheses. Life expectancy in OECD countries is associated with all seven categories we found, and particularly with health care expenditure, health financing policies, elements of medical care, health habits and population health, social determinants of health, social spending plus other external factors. In particular, in search of positive correlations to enhance a country’s life expectancy, our model suggests focusing on the full set of variables, rather than a single one. Indeed, the curvilinear projected trend we found for some variables (which have a maximum inside their range of variation) suggests combined and calibrated actions to maximise the global association and limit the cost to intervene. Some explanatory variables, such as the per capita healthcare expenditure, the density of physicians or hospital beds, are related to the cost of the health or social care system, which remain two important drivers through which policy makers could intervene shaping the relationship with population life expectancy by leveraging the level of public funds and their allocation. Conversely, other variables, also important in our model, related to GDP level, population size or population behaviour, are often reluctant to be controlled. Campaigns for control of calorie intake or for smoking prevention or reducing alcohol consumption take time to get the expected results. GDP level is quite difficult to change in the short period and similarly population size. Further variables, like climate-related ones, are scarcely governable, leading countries to spend budgeted resources elsewhere. The overall picture is a complex puzzle where longer life could be achieved by carefully fitting each puzzle piece to the others.

This research has a few limitations. First, although we paid attention to reverse causality, this could not be excluded completely. Second, data were collected for OECD countries, including most developed countries but excluding the majority of developing ones, thereby limiting the possibility of generalising results out of non-OECD countries. Third, we extracted data from databases of trusted organizations (e.g., World Bank, OECD) but, not having managed the full process of data acquisition, we cannot exclude errors in data collection. Fourth, our variable selection phase was influenced by the availability of data over our study period; therefore, a few variables potentially relevant for our study could not be taken into account.

Our analysis describes the situation of an average OECD country. We plan to expand our research by considering non-OECD countries, particularly developing ones. We are also considering investigating this topic further by looking at some sub-samples separately, singling out geographical areas where we can focus more in depth on their health coverage systems. In addition, the number of country/year observations could be increased, including earlier periods in the analysis finding other trusted sources of data. Since we expect a strong decrease in life expectancy with the COVID-19 pandemic period, our analysis should be repeated also considering more recent data.

In summary, our research supports the evidence that in OECD countries health expectancy has a correlation with many variables considered in our study, some of which addressed by policy maker decisions. Health care expenditures (per capita health care expenditure) together with health financing policies (out-of-pocket expenditure over total health expenditure) are positively associated with life expectancy. Furthermore, a greater supply of elements of medical care (physicians and hospital beds) are positively correlated with population longevity (life expectancy) as well as social determinants (share of population economically active) or health habits and population health (alcohol consumption and chronic respiratory diseases). We also found in our empirical analysis a negative relationship between population size and life expectancy: countries with large or growing populations must pay attention to living and health care conditions.

Politicians and policy makers should carefully consider the evidence of this study in light of the progressive reduction of the state budget that seems to be an unstoppable trend in Western economies before the pandemic COVID-19 period.

## Appendix

See Table 6

**Table 6:** Benchmark results (standardised coefficients)

	(1)	(2)	(3)	(4)	(5)
Timing of the regressors	Contemporaneous	1–year lag	3–year lag	5–year lag	Contemporaneous
Model	Fixed-effect	Fixed-effect	Fixed-effect	Fixed-effect	Arellano-Bond
H_EXP_T	3.237***	2.498**	2.364*	4.385***	2.940***
	(1.129)	(1.178)	(1.221)	(1.176)	(0.490)
(H_EXP_T)^2^	− 3.343***	− 2.570**	− 2.438**	− 4.295***	− 2.936***
	(1.141)	(1.193)	(1.237)	(1.201)	(0.497)
H_EXP_O	0.377***	0.366***	0.267**	0.135	0.398***
	(0.099)	(0.103)	(0.103)	(0.099)	(0.042)
PHYSIC	0.364*	0.492**	0.254	0.047	0.391***
	(0.191)	(0.219)	(0.229)	(0.239)	(0.082)
(PHYSIC)^2^	− 0.382**	− 0.572***	− 0.363	− 0.090	− 0.396***
	(0.166)	(0.203)	(0.222)	(0.242)	(0.071)
HOS_BED	− 0.761***	− 0.353	− 0.594	− 0.913**	− 0.010
	(0.278)	(0.330)	(0.365)	(0.394)	(0.136)
(HOS_BED)^2^	1.006***	0.664**	0.857**	1.027**	0.346***
	(0.246)	(0.299)	(0.348)	(0.404)	(0.120)
CAL	0.034	0.017	0.294***	0.318***	0.054
	(0.076)	(0.081)	(0.087)	(0.085)	(0.034)
ALC	− 0.395***	− 0.265***	− 0.100	0.066	− 0.415***
	(0.082)	(0.087)	(0.088)	(0.083)	(0.036)
RESP	− 0.378***	− 0.516***	− 0.339**	− 0.142	− 0.679***
	(0.120)	(0.133)	(0.142)	(0.142)	(0.062)
LAB_FOR	0.373***	0.349***	0.344***	0.294***	0.390***
	(0.074)	(0.078)	(0.085)	(0.094)	(0.033)
SOC	0.360***	0.465***	0.495***	0.319***	0.471***
	(0.094)	(0.097)	(0.099)	(0.093)	(0.042)
GDP_Q1	− 0.308***	− 0.390***	− 0.376***	− 0.333***	− 0.326***
	(0.094)	(0.095)	(0.093)	(0.088)	(0.040)
GDP_Q5	0.016	0.057	0.014	− 0.039	− 0.144***
	(0.106)	(0.115)	(0.121)	(0.117)	(0.048)
TEMP	− 0.006	0.053	0.426**	0.285	0.036
	(0.203)	(0.214)	(0.210)	(0.194)	(0.087)
POPUL	− 4.408***	− 5.408***	− 6.437***	− 6.976***	− 3.416***
	(0.962)	(1.016)	(1.111)	(1.240)	(0.415)
Constant	76.600***	76.677***	77.037***	77.788***	
	(0.120)	(0.136)	(0.133)	(0.120)	
Year effects	YES	YES	YES	YES	YES
F-Test health expenditures = 0	[0.013]	[0.098]	[0.141]	[0.001]	[0.000]
F-Test physicians = 0	[0.044]	[0.005]	[0.064]	[0.770]	[0.000]
F-Test hospital beds = 0	[0.000]	[0.000]	[0.000]	[0.032]	[0.000]
Within-group R-squared	0.909	0.906	0.900	0.902	
Mean VIF	2.53	2.52	2.60	2.61	2.53
Countries	36	35	35	35	35
Observations	601	550	481	415	550

The dependent variable is LIF_EXP_T. The model is fixed-effect OLS in Columns (1)–(4) and fixed-effect Arellano–Bond in Column (5). Coefficients are standardised. Standard errors in round parentheses; p values in squared parentheses. ***p < 0.01, **p < 0.05, *p < 0.1

## Footnotes

1 The eighteen countries are: Australia, Austria, Belgium, Canada, Denmark, Finland, France, Germany, Italy, Japan, the Netherlands, Norway, Portugal, Spain, Sweden, Switzerland, the United Kingdom and the United States.

2 The countries are: Australia, Austria, Belgium, Canada, Chile, Czech Republic, Denmark, Estonia, Finland, France, Germany, Greece, Hungary, Iceland, Ireland, Israel, Italy, Japan, Korea, Latvia, Lithuania, Luxembourg, Mexico, Netherlands, New Zealand, Norway, Poland, Portugal, Slovak Republic, Slovenia, Spain, Sweden, Switzerland, Turkey, United Kingdom, and United States. From the list of OECD countries we exclude only Colombia, for which we do not have data on social spending, and Costa Rica, which became an OECD member only in 2021.

3 The fit remained very high also when removing the year effects. Indeed, a model excluding the year variables obtained a within-group R-squared as large as 0.78.
